# Extended Longevity of Reproductives Appears to be Common in *Fukomys* Mole-Rats (Rodentia, Bathyergidae)

**DOI:** 10.1371/journal.pone.0018757

**Published:** 2011-04-13

**Authors:** Philip Dammann, Radim Šumbera, Christina Maßmann, André Scherag, Hynek Burda

**Affiliations:** 1 Department of General Zoology, Institute of Biology, University of Duisburg-Essen, Essen, Germany; 2 Department of Zoology, Faculty of Science, University of South Bohemia, České Budějovice, Czech Republic; 3 Central Animal Laboratory, University of Duisburg-Essen Medical School, Essen, Germany; 4 Institute for Medical Informatics, Biometry and Epidemology, University of Duisburg-Essen Medical School, Essen, Germany; Cajal Institute, Consejo Superior de Investigaciones Científicas, Spain

## Abstract

African mole-rats (Bathyergidae, Rodentia) contain several social, cooperatively breeding species with low extrinsic mortality and unusually high longevity. All social bathyergids live in multigenerational families where reproduction is skewed towards a few breeding individuals. Most of their offspring remain as reproductively inactive “helpers” in their natal families, often for several years. This “reproductive subdivision” of mole-rat societies might be of interest for ageing research, as in at least one social bathyergid (Ansell's mole-rats *Fukomys anselli*), breeders have been shown to age significantly slower than non-breeders. These animals thus provide excellent conditions for studying the epigenetics of senescence by comparing divergent longevities within the same genotypes without the inescapable short-comings of inter-species comparisons. It has been claimed that many if not all social mole-rat species may have evolved similar ageing patterns, too. However, this remains unclear on account of the scarcity of reliable datasets on the subject. We therefore analyzed a 20-year breeding record of Giant mole-rats *Fukomys mechowii*, another social bathyergid species. We found that breeders indeed lived significantly longer than helpers (ca. 1.5–2.2fold depending on the sex), irrespective of social rank or other potentially confounding factors. Considering the phylogenetic positions of *F. mechowii* and *F. anselli* and unpublished data on a third *Fukomys*-species (*F. damarensis*) showing essentially the same pattern, it seems probable that the reversal of the classic trade-off between somatic maintenance and sexual reproduction is characteristic of the whole genus and hence of the vast majority of social mole-rats.

## Introduction

**Figure 1:**
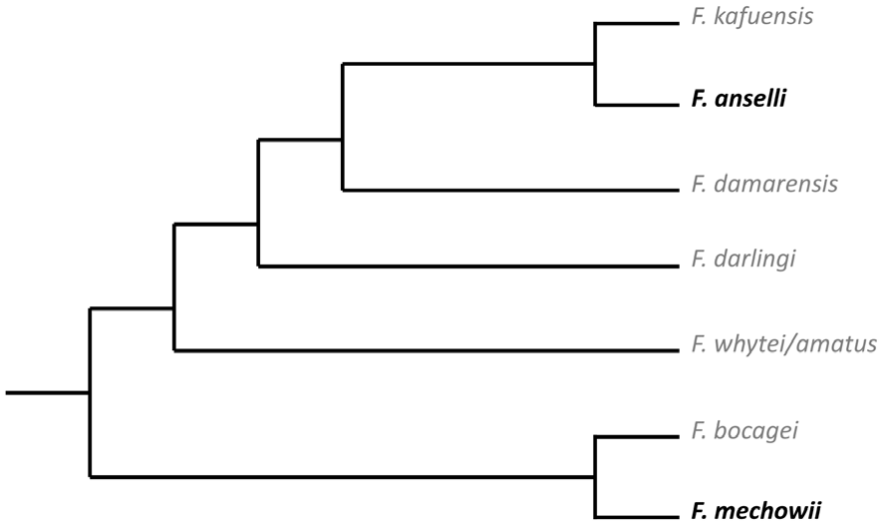
Phylogeny of the genus *Fukomys*. Combined phylogeny based on TTR Intron I, 12sRNA and cyt *b* sequences; adopted from [27] and [28].

Sexual reproduction is costly and often traded off against somatic maintenance and thus longevity [1], [2]. Striking exceptions are eusocial insects like hymenopterans (e.g. ants, bees, wasps) and termites [3], [4], where reproductive individuals live significantly longer than non-reproductive helpers. A few years ago, the same pattern was revealed for the first time in a mammal, namely the highly social Ansell's mole-rat *Fukomys anselli*: breeders of both sexes lived on average roughly twice as long as non-breeders and reached remarkable maximum lifespans of >20 years [5]. *Fukomys anselli* is a representative of the Bathyergidae, an African subterranean rodent family containing six genera. Three of them exhibit high degrees of sociality and reproductive skew: they live in multigenerational families in which only few individuals (often just a single founder pair) reproduce. Their adult offspring help to forage and store food and to extend, maintain and protect the burrow system. In the confines of their natal family, they assist in raising their younger siblings but forego own reproduction. It has been speculated that “the social mole-rats [in general], like some eusocial insects […], have evolved mechanisms that defer senescence in breeding females” [6]. This is an important claim, since if true, there would be ample opportunities to study the proximate mechanisms (e.g. endocrinology, gene expression patterns, epigenetics etc.) triggering divergent ageing rates within the same genotypes without the inevitable limitations of inter-specific comparisons, e.g. between fast and slow-ageing species. However, social or breeding status-dependent longevities have not been analyzed systematically either in other mole-rats except *F. anselli* or in any other cooperatively breeding mammal so far. Even in the naked mole-rat *Heterocephalus glaber* which has become an important ageing model in recent years [7], [8] on the ground of its extraordinary longevity per se (max. lifespan >30y [9]), the question of potential intraspecific, status-dependent differences in ageing rates has not been seriously addressed in this species to date. It is therefore not known whether the pattern found in *F. anselli* is observed in other social mole-rats (or cooperatively breeding vertebrates from other taxa), and if so, how great the effect is. Reliable datasets of additional species are therefore urgently required. This prompted us to analyze our 20-year breeding record of captive Giant mole-rats *Fukomys mechowii*, another social bathyergid species. Its mating system, social structure, and reproductive biology appear to match those of *F. anselli* and other *Fukomys*-species studied thus far [10]–[16]. Moreover, *F. mechowii* represents an ancient split within the *Fukomys* clade, whereas *Fukomys anselli* belongs to a much younger radiation ([17], [18]; (Fig. 1). Hence these two species encompass most of the phylogenetic range of the genus, which is by far the most speciose genus within the family Bathyergidae [19].

## Results

### Survival of breeders vs. non-breeders

**Figure 2:**
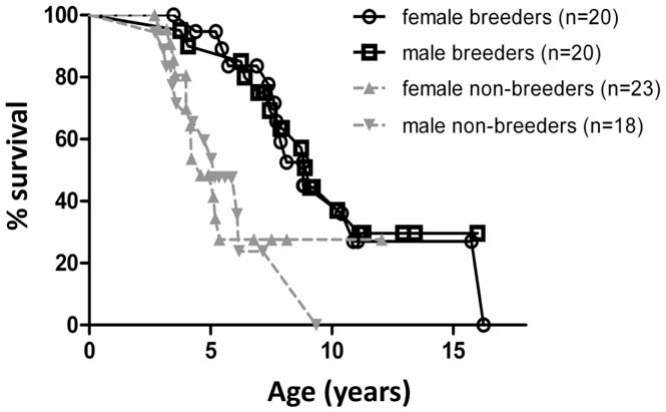
Survival curves of breeding and non-breeding *Fukomys mechowii*. Breeders lived significantly longer than non-breeders (log rank test, df = 1, χ^2^ = 15.49, *P*<0.001). There was no difference between males and females in either reproductive group (breeders ♀♀ vs. ♂♂ df = 1, χ^2^ = 0.21, *P* = 0.65; non-breeders ♀♀ vs. ♂♂ df = 1, χ^2^ = 0.01, *P* = 0.92).

**Figure 3:**
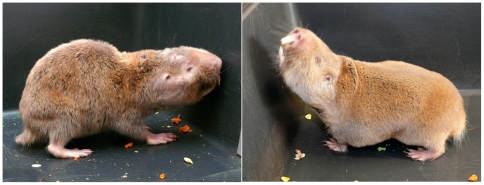
Breeding status determines the pace of aging in *Fukomys mechowii*. Left: An unusually old (∼9y) non-reproductive Giant mole-rat, showing obvious signs of senescence. Right: his mother (>15y), a breeding individual since 1996. Photos were taken at the same day (M. Schmitt).

Breeders lived significantly longer than non-breeders (log rank test, df = 1, χ^2^ = 15.49, *P*<0.001). There was no difference between males and females in either reproductive group (breeders ♀♀ vs. ♂♂ df = 1, χ^2^ = 0.21, *P* = 0.65; non-breeders: ♀♀ vs. ♂♂ df = 1, χ^2^ = 0.01, *P* = 0.92; Fig. 2). Amongst breeders, wild-caught individuals (7 ♀♀, 4 ♂♂) and breeders born in captivity (13 ♀♀, 16 ♂♂) did not show statistically different longevities in either sex (♀♀: *P* = 0.148; ♂♂: *P* = 0.975; not depicted). The oldest breeding males and females were >16 years and still alive by the end of data collection, and 11 out of 40 individuals (27.5%) had reached ages >10 years (cf. Fig. 3 for an example). By contrast, only one out of 41 non-breeders (2.4%) grew older than 10 years. Mean survival ± SE was 10.23y±1.09y and 8.83±0.56y for ♀♀ and ♂♂ breeders, and 4.56±0.19y and 5.74±0.64y in ♀♀ and ♂♂ non-breeders respectively. This corresponds to an extension of mean lifespan of breeders compared to non-breeders by around 54% in males and as much as 124% in females.

### Reproductive effort and survival of female breeders

Various standardized parameters of reproductive effort (number of litters/pups/weanlings or duration of lactation per 2 years, etc.; Table 1) were calculated for each breeding female in order to test whether they affected the female's life expectancies. Cox regression models could not identify significant effects of any of these parameters (singly or combined) on the breeding females' survival (Table 1).

**Table 1:** Reproductive effort and survival in breeding females (n = 20).

variable [unit]	mean ± SD [on original scale]#	hazard ratio [95% CI]	*P*-value
number of litters [n]	5.23±1.88	0.85 [0.56…1.29]	0.44
number of pups [n]	14.07±6.86	0.94 [0.86…1.04]	0.23
number of fully weaned [n]	3.70±3.39	0.84 [0.68…1.04]	0.10
lactation a [100 days]#	423±354 days	0.85 [0.69…1.04]	0.11
litter size [n]	2.57±0.70	0.61 [0.25…1.48]	0.27
interbirth interval [100 days]#	165±94 days	0.83 [0.35…2.00]	0.68
age at pairing [100 days]#	933±345 days	1.06 [0.85…1.31]	0.63
combined effort score [1 unit]	3.43±2.41	0.86 [0.67…1.12]	0.28

Univariate Cox analyses: hazard ratio point estimates and 95% confidence intervals (CI) and p-values (2-tailed) from Wald tests are reported. Null hypothesis (no effect): hazard ratio = 1. Ratios <1 indicate positive, >1 negative effects of a given parameter in units of the respective variable on survival. Except age at pairing, all parameters refer to a defined period of time (the first two years after delivery of the first litter) in order to standardize the data.

a lactation effort defined as: number of sucklings * their respective lifespan before weaning at approx. 90 days [10].

# Note that the units of some variables differ between the descriptive (mean ± SD) and the inferential illustration in the cox model. This transformation was necessary to better display the hazard ration point estimates.

### Effect of social status on survival

**Figure 4:**
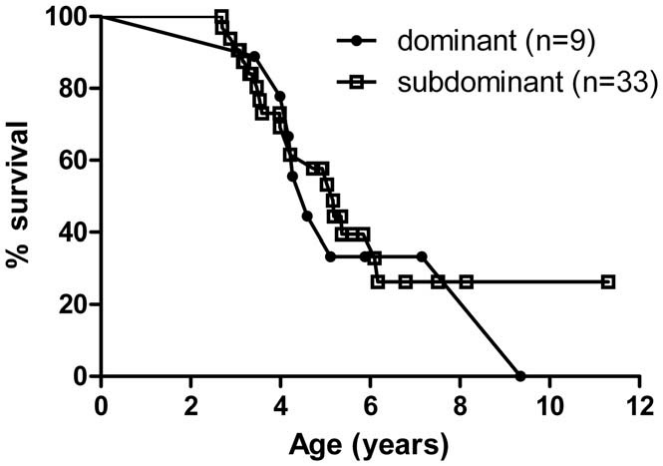
Survival of non-breeders growing up in different social environments. Sexes combined. Survival probabilities did not differ between dominant (early-born) and subdominant (late-born) non-breeders (log rank test, df = 1, χ^2^ = 0.05, *P* = 0.82).

Social rank did not seem to affect survival probabilities: first-born “dominant” non-breeders (n = 9) did not live longer or shorter than their respective younger siblings, “subdominant” non-breeders (n = 33) (log rank test, df = 1, χ^2^ = 0.05, *P* = 0.82; Fig. 4). Modifying criteria for group assignment (i.e. the number of higher-ranking siblings allowed in each group), or analysing genders separately, produced essentially the same result (not shown).

### Activity patterns

**Figure 5:**
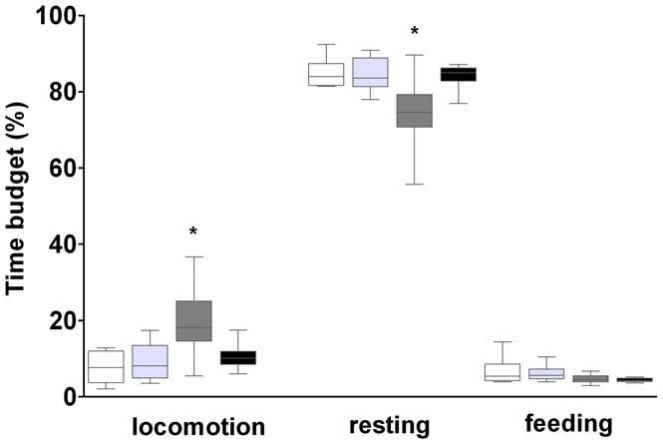
Activity data based on 72 h-observations. Box plots showing the time budgets allocated to locomotion, food intake and resting. All behaviours are expressed as % of time spent per 72 h-observation. Group sizes were n = 6 for each breeder group and n = 12 for each non-breeder group, respectively. Colour codes (from left to right for each behaviour): white = female breeders, light grey = male breeders, dark grey = female non-breeders, black = male non-breeders. Boxes represent the interquartile range, bars within boxes are median values and whiskers indicate the 5th and 95th percentile. Significant differences denoted by asterisks.

Analysis of daily activity patterns identified no between-group differences regarding the time allocated to feeding (two-way ANOVA with factors sex and breeding status: F_3,32_ = 2.76, *p*>0.05), but it did show significant differences amongst groups regarding locomotion (F_3,32_ = 8.32, *p*<0.05) and resting (F_3,32_ = 7.37, *p*<0.05). The analysis of main and interaction effects revealed that female non-breeders rested less (74.49±2.47%) and showed more locomotor activity (19.68±2.39%) than the three other groups, while no differences in these behaviours were found among the latter (*resting*: ♀♀ breeders 84.86±2.03%, ♂♂ breeders 84.44±1.87%, ♂♂ non-breeders 83.79±0.92; *locomotion*: ♀♀ breeders 7.69±2.07%, ♂♂ breeders 9.09±2.06%, ♂♂ non-breeders 10.77±0.97%; all behaviours expressed as mean ± SE of time spent per 72 h – observation; Fig. 5).

## Discussion

Apparently, divergent intrinsic ageing rates exist in *F. mechowii* that are mainly or exclusively determined by the reproductive status of the animals. As in *F. anselli*, breeders of both sexes outlive their non-reproductive counterparts by far, with reproductive individuals living significantly longer than helpers. Female breeders, despite their investment into long pregnancy and lactation [10], even live more than twice as long as same-sex non-breeders.

The fact that the breeder group contained animals born in the wild (where age cannot be determined) implies that the true lifespan of breeders may be larger than reported here. In contrast, all non-breeding data came from laboratory-raised individuals whose lifespans could be recorded accurately. This inhomogeneity also implies that there might have been less genetic diversity in non-breeders than the breeding groups. Theoretically, this could have flawed our data to a certain extent due to inbreeding effects. This, however, is unlikely as *i*) wild-caught breeders did not show different longevities than lab-raised breeders in either sex and *ii*) breeders continued to have significantly higher longevities than non-breeders even after wild-caught breeders were excluded from the analysis (i.e. both the breeder and the non-breeder group consisted only of lab-raised animals).

Social stress given by the dominance hierarchies within the families does not seem to be the driving force for these differences either, as social rank does not affect survival probabilities in non-breeders alone, i.e. when the effect of reproductive status is eliminated. Other lines of evidence corroborate the assumption that social rank has little to do with ageing in *F. mechowii*: in captive colonies of this species, there is no competition for mating partners, and hardly any for food. Overall aggression levels are low, and sociopositive behaviours (grooming, huddling, communal sleeping, etc.) are prevalent. Consequently, there is little reason to believe that subordinate individuals suffered considerable social stress. On the basis of all these factors, we conclude that social rank is not a likely cause of the observed differences in lifespan. We are aware, however, that this assumption needs to be further corroborated by endocrinological data. We are also aware that the situation may be somewhat different in the wild. It is important to point out, however, that our finding of different longevities between breeders and non-breeders was made in captivity. For an appropriate discussion of potential caveats or artefacts on the proximate level, it is therefore necessary to analyze intrinsic and extrinsic factors characterizing way of life of captive (and not wild) animals likewise.

Activity patterns and ageing schemes of the four groups under investigation did not correlate: the “slow-ageing” breeders (both sexes) and the “fast-ageing” male non-breeders showed indistinguishable activity budgets. Female non-breeders were more active and rested less than all other groups, yet they aged at the same pace as the less active male non-breeders. Thus, although it cannot be excluded that higher activity imposes a metabolic cost to female non-breeders and even affects their ageing rate to a certain extent (on account of the enhanced production of reactive oxygen species due to increased metabolic rates), group-specific differences in locomotor activity can hardly explain the dramatic divergence of group-specific survival curves in our captive families of this species, at least not on a simple “wear and tear”-basis.

It is also unlikely that a priori differences in intrinsic quality between breeders and non-breeders – which theoretically could result from an unintended bias towards high-class individuals during the selection of breeders – produced the divergent ageing rates: our pairing scheme is purely arbitrary and determined by the availability of unfamiliar mates of similar age at any given time rather than by any other factor.

Our findings demonstrate that the unusual ageing pattern, which was previously only known in *F. anselli* among vertebrates, is not just an oddity of this species, but extends to at least one other social mole-rat species as well. This is probably an underestimation, because in *F. damarensis*, another well-studied *Fukomys*-species, breeders appear to outlive the non-breeders, too (N. Bennett, personal communication). Given that within the genus *F. mechowii* is only distantly related to *F. anselli* (Fig. 1) and that all *Fukomys* species are believed to be relatively uniform as far as their general behavioural ecologies are concerned [14]–[16], we believe that role-dependent ageing is most probably characteristic of the entire genus *Fukomys*. The pronounced benefit of breeding for longevity seen in this genus is unparalleled in any other vertebrate group studied thus far and greater than most experimental interventions that extended lifespan in vertebrates, such as caloric restriction [20] or diets containing resveratrol or rapamycin [21], [22]. It is worth mentioning that *Fukomys* contains by far the most species of all six genera that constitute the family Bathyergidae [17]–[19]. Opportunities to study such highly divergent ageing rates within the same genotypes hence are much more ample than previously thought.

Whether the reported ageing pattern can be even further generalized to the other two social bathyergid genera (*Cryptomys* and *Heterocephalus*) is unclear. The results obtained in *Fukomys sp.* reveal some differences to the ageing pattern of naked mole-rats *Heterocephalus glaber* in that the latter's breeders and non-breeders do not differ in maximum lifespan [23], [7]. However, this does not necessarily mean that breeding status does not affect ageing in *H. glaber* at all. Maximum lifespan is not an appropriate measure of (average) life expectancy, and status-specific survival curves have not yet been published for *H. glaber*. In the wild, breeders have been reported to live ∼4-fold longer than non-breeders (Braude, personal communication cited in [24]). While this observation, which is again based on estimates of maximum lifespan derived from capture-recapture data, can be interpreted as an effect of higher extrinsic mortality risks faced by non-breeders [25] and/or their dispersal from the natal colonies [14], [24], the existence of (additive?) differences in intrinsic ageing rates between breeders and non-breeders cannot be excluded unless formal demographic analysis are available. Given the extensive lab data which is available for this species, this should be easy to rectify.

The proximate mechanisms that trigger the divergent ageing rates of breeders and non-breeders in *Fukomys* sp. are completely unknown at the moment. Interestingly, in *F. mechowii*, we could not identify single or combined components of reproductive activity as being beneficial or detrimental to the breeding females' survival (Table 1). One lesson to learn from this is that reproductive females which invest more into breeding do not seem to pay an apparent cost of reproduction (as is commonly seen in other vertebrates, e.g. [2]; but see [26] for captive animals). On the other side, it remains enigmatic which components of reproductive activity actually trigger the apparent retardation of ageing induced by attainment of breeder status. Our sample size may still be too small to draw definite conclusions on this matter, but at this stage it seems likely that no single behaviour alone retards or accelerates the pace of ageing in mole-rats. One may speculate that pair bonding and onset of sexual activity rather triggers some sort of categorical switch in gene expression patterns, probably acting on multiple somatic systems which together lead to the substantial lifetime extension experienced by mole-rat breeders.

Alternatively, non-breeding could simply be more stressful than breeding, at least under the benign conditions of a sheltered laboratory environment. Adult non-breeders are functionally and physiologically fully mature and capable of reproduction; in our laboratory, single copulation events induced by short-term-pairing of unfamiliar non-breeders have repeatedly led to pregnancy and the delivery of healthy litters, no matter whether the mates stayed together as a new pair or whether they were returned into their natal colonies after copulation ([12], own unpublished data). Although speculative at the moment, we do not want to rule out that a continued lack of mating opportunities might stress the animals and lead to impaired homeostasis in the long term. Also, sexual activity and pair bonding enjoyed by the breeding pair, but not by the non-breeders, is expected to alter the levels of various hormones and neurotransmitters, some of which (e.g. oxytocin) have been linked to enhanced immunity, reduced risk of cardiovacular diseases and reduced stress effects [27], [28]. These hypothesis are mutually non-exclusive and could well act in concert to explain the divergent ageing rates in *Fukomys* sp. Studying the underlying mechanisms (gene expression, endocrinology etc.) in detail would be a logical next step and could yield fascinating insights into the biology of mammalian ageing.

Although the body of information on the biology of bathyergids in their natural habitats is constantly growing, we still know relatively little about the exact age composition, dispersal patterns and subdivision of labour in wild-ranging mole-rat families. Transferring our results from the laboratory to the conditions in the wild should therefore be done with caution at this stage. However, patterns of residency in the wild (where breeders of Damaraland mole-rats and naked mole-rats could be recaptured for several years longer than non-breeders [29], [30]) are at least not in disagreement with the ageing patterns reported here. It will therefore be interesting to cross-check our results in the wild, e.g. with the help of additional long-term capture-recapture studies, telemetry or the use of appropriate ageing biomarkers applied to wild families. In eusocial hymenopterans, caste-specific differences in extrinsic mortality are considered the ultimate cause for the divergent ageing rates of queens and workers [3], [31]. For example, ant queens (which age slowly) often spend their whole reproductive lives in sheltered nests, while outside foraging workers are at greater risk due to predation and hence age faster [31]. In accordance with this, caste-specific ageing rates do not seem to differ in some termite species where workers never leave their nests (and therefore face the same extrinsic mortality risk as the reproductives) and where reproductives can be replaced by workers after their death [32]. In *Fukomys*, most tasks are performed underground, suggesting that extrinsic mortality risks of breeders and non-breeders are roughly the same under normal circumstances. However, it cannot be excluded that in the wild, non-breeders might be more prone than breeders to fulfil high-risk tasks such as burrowing near the surface, or nest defense ([14] and own unpublished data). Moreover, non-breeders in *Fukomys* usually need to disperse to reproduce on their own, as incest avoidance prevents replacement of lost breeders from within. Dispersal is risky, but becomes irrelevant once a mate is established. Shall differential extrinsic hazards have ultimately shaped the bimodal lifespan patterns in *Fukomys*, then one of these factors (differential tasks, dispersal) or a combination of both could be the cause.

It might be worth also looking at potentially role-dependent ageing rates in other cooperatively breeding vertebrates. Many components of the mole-rats' social and mating system (e.g. reproductive skew, philopatry of helpers, incest avoidance, within-group levels of social stress) are shared by other cooperatively breeding species, yet each to varying degrees. This opens up great opportunities for elucidating the impact of these components on vertebrate ageing. Sharp & Clutton-Brock have recently published one of the very few studies on senescence patterns in natural populations of a cooperative breeder, the meerkat *Suricata suricatta* [33]. Their paper focused on reproductive senescence in breeding females (which they were able to demonstrate), but they also provided data which suggested that there might be role-dependent survival probabilities in meerkats, too, with breeders outliving non-breeders just as is the case for mole-rats (see figure 1 in [33]). A thorough analysis of this phenomenon and its underlying factors will further improve our understanding about how ageing and survival probabilities are modified by the co-existence of breeders and helpers in cooperatively breeding species. Given the large ecological and behavioural diversity amongst cooperative breeders, and their multiple phylogenetic origins [34], [35] we predict that divergent ageing rates of reproductives vs. helpers are not a universal feature of cooperative breeders, but depend strongly on at least two main factors: *i*) future reproductive options of non-breeding helpers, and *ii*) potential differences in extrinsic mortality between breeders and helpers. Divergent ageing rates should be less likely, or less pronounced, in societies in which helpers can attain breeder status within the colony (e.g. via inbreeding or queuing) and more likely (pronounced) in species where dispersal is indispensible to an individuals' own reproduction, especially if dispersal is risky and good conditions for successful dispersal are rare and unpredictable. Status-specific ageing rates are also more likely to differ if breeders and non-breeders have differing exposure to extrinsic hazards and less likely if they do not.

## Methods

### Study species

The giant mole-rat is the largest bathyergid species with social habits. Mean family size in giant mole-rat amounts to 12–13 and may reach over 20 animals ([36], [37], Šumbera et a al. under review). Adult males weigh on average almost 400 g, adult females >250 g [36]. As all other bathyergids, Giant mole-rats feed mainly on the storage organs of geophytes ([36], [38], Šumbera et al. under review]. They reside predominantly in mesic areas of Zambia, Angola, and Democratic republic of Congo [39] where they dig and maintain complex burrow systems with a tunnel length of up to >2,000 m (Šumbera et al. under review).

### Housing and breeding

We have kept *F. mechowii* in captivity since 1990, under conditions described in detail elsewhere [10]. Briefly, animals are kept as pairs or families in terrariums with horticultural peat. Food is provided *ad libitum*. Systematic breeding started using a stock of eight wild-caught animals which gradually proliferated over the years. Additional wild-caught specimens were introduced from various field trips between 1992 and 2007. To establish a family, adult non-breeders are removed from their families and paired with an unrelated opposite sex individual in a separate terrarium. Such new breeders retain breeding status until they or their partners die, with most of their offspring remaining in their natal colonies. Due to strict inbreeding avoidance, adult offspring remain reproductively quiescent unless paired with an unfamiliar mate. The reproductive division of labor is thus clear-cut in our stock, allowing assignment of each individual to either the “breeder” or “non-breeder” group.

### Survival of breeders vs. non-breeders

Paired animals with offspring entered the study as breeders. Their mean age at pairing was between 2–3 years (mean ± SE ♀♀: 993±94 days, ♂♂: 857±112 days). Unpaired animals older than these respective ages which remained in their natal colonies and did not reproduce entered the analysis as non-breeders. Animals used for mate choice or other experiments were excluded from analysis. Wild-caught animals that did not breed in captivity were also excluded because their reproductive status before capture was uncertain. Survival probabilities were calculated using the Kaplan Meier estimator of survival rates and compared using log rank tests. Individuals whose lifespan was followed until their death entered the study as complete observations. Individuals who were still alive in June 2010 were included as censored observations. Wild-caught animals were classified on the basis of fur coloration and weight as “juveniles” (50–100 g), “subadults” (100–200 g) or “adults” (>200 g) by the date of capture, and assessed as being a minimum of 3 months (juveniles), 6 month (subadults) or 12 month (adults) respectively. These are cautious estimates, and some specimens were probably older. Since wild-caught animals either became breeders or were excluded from analysis (see above), our calculations of longevity are conservative for the breeder group, but exact for the non-breeder group. Altogether, 81 animals entered the study (breeders: 20 ♀♀, 20 ♂♂; non-breeders: 23 ♀♀, 18 ♂♂)

### Reproductive effort and survival of female breeders

Life history theories of ageing usually assume a negative correlation between reproductive effort and longevity. This could still be apparent *within* the breeder groups even if they – as a whole - live on average longer than non-breeders. We were therefore interested whether the degree of individual reproductive effort had an effect on individual life expectancy in breeders. Because reproductive effort is hard to measure in males retrospectively, we focused on females to address this question.

There was huge variation amongst females regarding the duration of their reproductive lifespans (ranging from ca. 2–14 years after pairing). We therefore measured 7 reproductive variables (see Table 1) covering only the first two years of each female's reproductive tenure, thereby standardizing the data. We then computed univariate cox regression models for each variable. The advantage of using a cox regression model (as compared to the use of a log rank test only) is that the cox model allows for estimating an effect of a particular variable. To evaluate multivariable contributions in the relatively small sample, we summarized the 7 reproductive variables by creating an “combined effort score” for each female using median-splits for each variable to distinguish between “high-effort females” (score 1) and “low-effort females” (score 0; if the respective variable measurement was unknown or identical to the median the score was set to 0.5). The “combined effort score” for each female was then calculated as the sum across all 7 variable scores. Hence, this combined score could have ranged between 0–7 with higher values being indicative of higher reproductive effort of the particular female. The combined effort score may be regarded as a latent variable, thus potentially having greater statistical power to detect an effect as compared to the single measurements. It was also analysed by cox regression. Note that we did not include measures of lifetime reproductive success (e.g. total number of pups per lifetime) because such measures could lead to a confusion of effect and cause: long-lived females have more time to produce offspring, almost necessarily creating a positive correlation between measures of lifetime reproductive success and longevity. They also would have caused problems to include individuals which were still alive at the end of the study.

### Determination of social rank and its potential effect on life expectancy

There is a social hierarchy in Giant mole-rat families, and since breeders are the founders of their colony, they are the oldest and among the top-ranking individuals in their respective families. Social rank is known to influence longevity in some species, therefore its uneven distribution amongst mole-rats (always high in breeders, variable in non-breeders) might influence their group-specific life expectancies. To disentangle the potential effects of reproductive status and social rank on ageing, we excluded breeders from analysis and assigned each non-breeder to one of two groups: a) “dominant” individuals that attained maturity when no dominant siblings were present; and b) “subdominant” individuals that attained maturity when one or more dominant siblings were present. This allocation is possible because in *F. mechowii* families, relative rank of each individual depends mainly on body mass ([11] and own unpublished data). Males become larger than females [10], thus fully grown males >3 years usually dominate adult females irrespective of the female's age. Within a given sex, birth order is a good proxy for the dominance pattern. It is therefore possible to estimate the social rank of each individual retrospectively if the actual colony composition at that time is considered. Again, survival probabilities of both groups were compared using Kaplan-Meier-statistics (see above).

### Activity budgets

6 breeders and 12 non-breeders of each sex were individually marked with fur colour (RAIDEX®, Germany) and filmed for 72 h. The time allocated to three different behaviours was quantified using the NOLDUS® Observer package XT 9.0: a) resting (i.e. showing no evident signs of activity over an extended period of time, usually in a well-defined communal nest); b) feeding; and c) locomotion (covering all evident activities except feeding, e.g. digging, nest building, transportation of young or food items, walking, running, etc.). Time budgets were compared using two-way ANOVAs with factors sex and breeding status.
